# HPV6-associated cervical squamous cell carcinoma: a case report and literature review

**DOI:** 10.3389/fonc.2026.1798759

**Published:** 2026-03-16

**Authors:** Zhenzhen Li, Hui Shen, Ting Hao, Cuncun Guo, Jie Zhang

**Affiliations:** 1 Department of Pathology, Shandong Provincial Maternal and Child Health Care Hospital Affiliated to Qingdao University, Jinan, Shandong, China; 2 Department of Pathology, Dingtao District People’s Hospital, Heze, Shandong, China; 3 Department of Pathology, Shandong Cancer Hospital and Institute, Shandong First Medical University & Shandong Academy of Medical Sciences, Jinan, Shandong, China

**Keywords:** cervical squamous cell carcinoma, HPV6, low-risk human papillomavirus, molecular profiling, TERT promoter, TP53 mutation

## Abstract

Low-risk human papillomavirus (HPV) types are traditionally regarded as non-oncogenic and are primarily associated with benign epithelial proliferations. Invasive cervical squamous cell carcinoma (CSCC) related to low-risk HPV infection is exceedingly rare. Here, we report a rare case of HPV6-associated CSCC in a postmenopausal woman and comprehensively characterize its clinicopathological, immunophenotypic, and molecular features, with a review of the relevant literature. The patient presented with irregular vaginal bleeding and an exophytic cervical mass. Histopathological examination revealed verrucous papillary squamous epithelial hyperplasia with prominent fibrovascular cores, surface koilocytosis, marked cytological atypia, and focal superficial stromal invasion (≤ 3 mm). Immunohistochemical analysis demonstrated negative p16 expression and a high Ki67 proliferation index. HPV genotyping detected isolated low-risk HPV type 6, with no evidence of high-risk HPV infection. Targeted next-generation sequencing identified multiple somatic alterations, including pathogenic mutations in *TP53*, *CDKN2A, TERT* promoter, and *LATS1*, suggesting a host-driven molecular carcinogenic process. The present study supports an indirect carcinogenic pathway for low-risk HPV–associated cervical squamous cell carcinoma, in which persistent viral-induced epithelial proliferation may facilitate the accumulation of oncogenic host mutations. Recognition of this rare entity expands the pathological spectrum of HPV-associated cervical cancer and underscores the importance of integrating morphology, immunohistochemistry, and molecular testing for accurate diagnosis and risk assessment.Awareness of this rare entity may help avoid underdiagnosis of malignant transformation in exophytic cervical lesions associated with low-risk HPV infection.

## 1 Materials and methods

### 1.1 Clinical data

A 55-year-old woman presented to the gynecology clinic of our hospital with a history of irregular vaginal bleeding for more than one month. No prior HPV testing or cervical cytology (Pap test) had been performed in this patient before presentation. Gynecological examination revealed an enlarged cervix with marked erosive changes and a friable, cauliflower-like exophytic mass measuring approximately 2 cm in diameter at the external cervical os, which bled easily on palpation. No abnormalities were detected in the uterine body or bilateral adnexa on bimanual examination.

Transvaginal ultrasonography demonstrated an enlarged cervix with a hypoechoic mass measuring 3.6 × 2.8 cm, characterized by ill-defined margins and heterogeneous internal echogenicity. Color Doppler flow imaging revealed punctate and strip-like intralesional blood flow signals. No abnormal findings were identified in the uterine cavity or bilateral adnexa.

Histopathological evaluation of a cervical biopsy specimen established a diagnosis of HPV6-associated papillary cervical squamous cell carcinoma. The patient subsequently underwent radical hysterectomy at another institution. Postoperative pathological examination revealed cervical squamous cell carcinoma with superficial stromal invasion (invasion depth ≤ 3 mm), without lymphovascular space invasion or lymph node metastasis.

### 1.2 Methods

#### 1.2.1 Histopathological examination

All biopsy and surgical specimens were fixed in 10% neutral buffered formalin for 24–48 hours, followed by routine dehydration, clearing, paraffin embedding, and serial sectioning at a thickness of 4 μm. Sections were stained with hematoxylin and eosin (H&E). Histopathological evaluation was independently performed by two experienced pathologists in a blinded manner to assess morphological features and establish the final diagnosis.

#### 1.2.2 Immunohistochemistry

Immunohistochemical staining was performed using the EnVision two-step method. Primary antibodies against P16 and Ki67 were applied according to the manufacturer’s instructions. Immunoreactivity for P16 was defined as nuclear and/or cytoplasmic brown granular staining, while Ki67 positivity was defined as brown granular nuclear staining. Semi-quantitative evaluation was based on the proportion of positive tumor cells and staining intensity.

#### 1.2.3 HPV Genotyping

HPV genotyping was performed on biopsy specimens using a polymerase chain reaction (PCR)–reverse dot blot assay, which is capable of detecting 23 HPV subtypes, including 17 high-risk types (HPV16, 18, 31, 33, 35, 39, 45, 51, 52, 53, 56, 58, 59, 66, 68, 73, and 82) and 6 low-risk types (HPV6, 11, 42, 43, 81, and 83). The procedure included HPV DNA extraction, PCR amplification of the conserved L1 gene region, hybridization with subtype-specific probes, and chromogenic detection. The absence of subtype-specific signals was interpreted as HPV negativity.

#### 1.2.4 Hybrid capture assay and next-generation sequencing

Additional molecular characterization of the tumor tissue from the biopsy was performed using hybrid capture-based HPV nucleic acid detection and next-generation sequencing (NGS). NGS was conducted using multiple sequencing platforms and covered 572 cancer-related genes at the DNA level and 2660 genes at the RNA level. The assay was designed to detect single-nucleotide variants, small insertions and deletions, gene fusions, homozygous deletions, and copy number alterations. Microsatellite instability status, tumor mutation burden, tumor immune microenvironment characteristics, gene expression profiles, and polymorphisms associated with chemotherapeutic drug metabolism were simultaneously assessed. All procedures were performed in accordance with standardized laboratory protocols and quality control requirements.

## 2 Results

### 2.1 Gross findings

#### 2.1.1 Biopsy specimens

The biopsy specimens consisted of multiple small tissue fragments with an aggregate size of approximately 1.0 × 1.0× 0.5 cm. The tissues were grayish-white, firm in consistency, and entirely submitted for histological examination.

#### 2.1.2 Radical resection specimens

The surgical specimen included the uterus with bilateral adnexa. A grayish-white, firm lesion measuring approximately3.0 × 3.0 cm with ill-defined margins was identified at the external cervical os. The myometrium, endometrium, bilateral fallopian tubes, and ovaries were grossly unremarkable.

### 2.2 Histopathological findings

#### 2.2.1 Biopsy specimens

**Figure 1:**
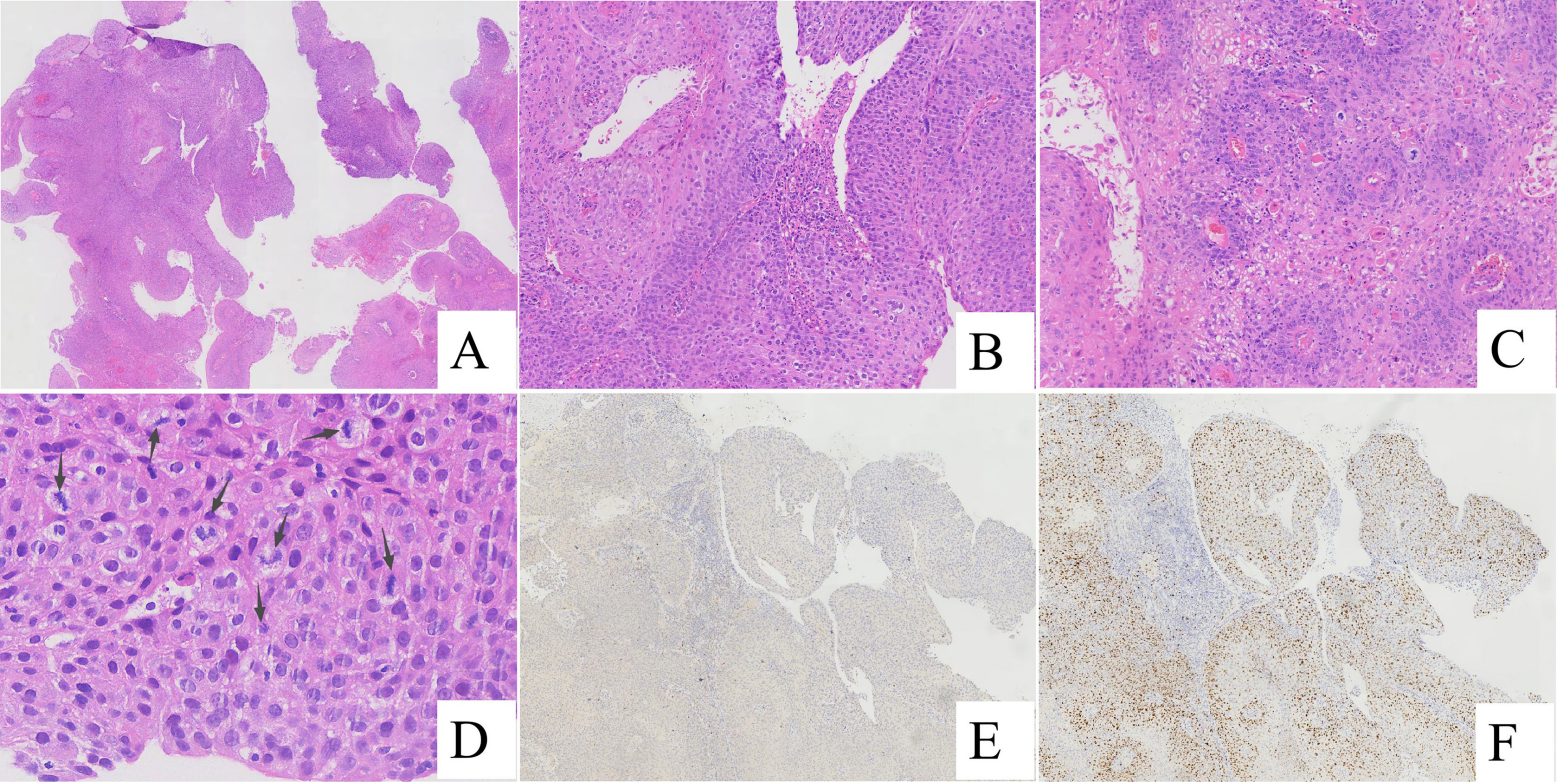
Biopsy specimens. **(A)** Papillary squamous epithelial hyperplasia with fibrovascular cores (H&E, ×20). **(B)** Full-thickness squamous epithelial atypia extending from the basal layer to the surface, with surface koilocytosis (H&E, ×100). **(C)** Papillary squamous epithelium with focal fusion and keratinization (H&E, ×100). **(D)** Frequent mitotic figures (arrows) (H&E, ×400). **(E)** Negative P16 immunostaining (EnVision method, ×40). **(F)** High Ki67 proliferation index (~60%) in hotspot areas (EnVision method, ×40).

Low-power examination demonstrated an exophytic verrucous papillary architecture composed of squamous epithelial proliferations with prominent fibrovascular cores. Focal papillary fusion and surface keratinization were observed. No unequivocal stromal invasion was identified in the biopsy specimens (**Figures 1A, B**).

At higher magnification, the surface epithelium showed characteristic koilocytosis. The squamous epithelium exhibited marked hyperplasia with significant cytological atypia exceeding that typically observed in benign condyloma acuminatum, accompanied by abnormal keratinization and readily identifiable mitotic figures (**Figures 1C, D**).

#### 2.2.2 Radical resection specimens

**Figure 2:**
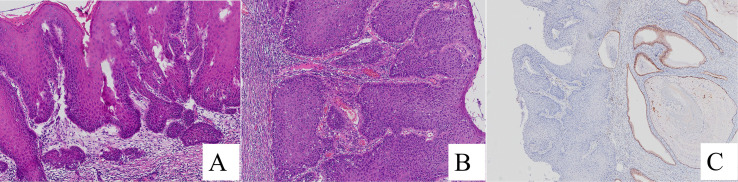
Radical hysterectomy specimens. **(A)** Superficial stromal invasion by well-differentiated exophytic papillary squamous epithelium with keratinization (H&E, ×100). **(B)** Marked cytological atypia (H&E, ×400). **(C)** Negative P16 immunostaining in the resection specimen (EnVision method, ×100).

Histological examination of the hysterectomy specimen revealed a predominantly exophytic papillary growth pattern. The surface epithelium showed dyskeratotic cells and characteristic koilocytes, along with marked squamous epithelial atypia and frequent mitotic activity.Foci of superficial stromal invasion were identified, accompanied by a stromal reaction, with the maximum depth of invasion being ≤ 3 mm (**Figures 2A, B**). No lymphovascular space invasion was observed.

### 2.3 Immunohistochemical findings

Immunohistochemical staining demonstrated negative P16 expression in both the biopsy and radical resection specimens (**Figures 1E**, **2C**). Ki67 immunostaining of the biopsy specimen showed a high proliferative index, with approximately 60% of tumor cells positive in hotspot areas (**Figure 1F**).

### 2.4 HPV detection results

In this study, PCR-reverse dot blot hybridization was used to perform HPV genotyping screening on biopsy tissue specimens. Low-risk HPV 6 was detected. All tested high-risk HPV subtypes and the remaining low-risk HPV subtypes were negative.

### 2.5 Final pathological diagnosis

Based on the evaluation of histopathology, immunohistochemical profile, and HPV genotyping results, the cervical biopsy specimen was diagnosed as HPV6-associated cervical squamous cell carcinoma.The radical specimen was diagnosed as HPV6-associated cervical squamous cell carcinoma with predominant exophytic growth and superficial stromal invasion (≤ 3 mm).

### 2.6 Next-generation sequencing results

Next-generation sequencing performed on the biopsy specimen identified nine somatic genetic alterations. These included two nonsense mutations in *TP53* [p.(Y126) and p.(S183)], a nonsense mutation in *CDKN2A* (c.238C>T), a missense mutation in *DNMT3A* (c.1913C>T), a promoter mutation in *TERT* (c.-124C>T), three mutations in *LATS1* (c.2648C>G, c.2726C>G, and c.1969C>T), and a synonymous mutation in *SPTA1* (c.4512A>G). Detailed mutation information and variant allele frequencies are summarized in **Table 1**.

**Table 1:** Next-generation sequencing (NGS) results.

Gene	Test result	Abundance/Copy number
*TP53*	exon5 c.378C>G p.(Y126*) NM_000546.6	3.74%
*TP53*	exon5 c.548C>G p.(S183*) NM_000546.6	3.55%
*CDKN2A*	exon2 c.238C>T p.(R80*) NM 000077.5	51.94%
*DNMT3A*	exon16 c.1913C>T p.(S638F) NM 022552.5	32.22%
*TERT*	FlankingRegion5 c.-124C>T NM 198253.3	33.60%
*LATS1*	exon6 c.2648C>G p.(S883*) NM_004690.4	33.99%
*LATS1*	exon6 c.2726C>G p.(S909C) NM 004690.4	34.69%
*LATS1*	exon4 c.1969C>T p.(R657C) NM 004690.4	27.90%
*SPTA1*	exon32 c.4512A>G p.(Q1504=) NM 003126.4	14.43%

## 3 Discussion

Cervical squamous cell carcinoma (CSCC) remains a major global health burden, with the majority of cases etiologically linked to high-risk human papillomavirus (HPV), particularly HPV16 and HPV18 (1–3). In contrast, low-risk HPV types are traditionally regarded as non-oncogenic and are primarily associated with benign epithelial proliferations (4–7). However, accumulating evidence suggests that under specific circumstances, low-risk HPV—especially HPV6—may contribute to malignant transformation (8). The present study reports a rare case of HPV6-associated CSCC and provides a comprehensive clinicopathological and molecular characterization, thereby expanding the biological spectrum of HPV-associated cervical carcinoma.

### 3.1 Pathological and immunophenotypic features

HPV6-associated CSCC typically presents as an exophytic, papillary, or verrucous cervical mass, sometimes resembling a cauliflower-like lesion. This growth pattern differs from the infiltrative morphology commonly observed in conventional CSCC and may lead to misdiagnosis as giant condyloma acuminatum (4, 8, 9). Previously reported lesions have ranged in size from 2 to 4.5 cm, with a median size of approximately 4.0 cm, which is consistent with the findings in the present case. Clinical information is summarized in **Table 2**.

**Table 2:** Reported cases of low-risk HPV–associated cervical squamous cell carcinoma.

Case	Author (Year)	Age (years)	Clinicalpresentation	HPV (PCR)	Tumor size (cm)	Growth Pattern	p16	p53	Ki67	Key molecular alterations	Follow-up (months)	Outcome
1	Masuda et al. (2018) (9)	43	Long history of LSIL	HPV6	4.0	Exophytic, Prominent stromal invasion	Negative	positivity 50–60%	22.1%	Not reported	12	LN & lung metastasis
2	Liu et al. (2019) (4)	50	Vaginal bleeding, cervical mass	HPV6	NA	Papillary SIL with superficial invasion	Negative	Strong and diffuse	Ki-67 was significantly elevated	*TP53* mutation	NA	NA
3	Liu et al. (2019) (4)	51	NA	HPV6	NA	Papillary SIL with superficial invasion	Negative	Strong and diffuse	Ki-67 was significantly elevated	Negative	NA	NA
4	Rokutan-Kurata et al. (2020) (26)	43	history of (CIN) 1-2	HPV6	NA	Exophytic,Prominent stromal invasion	Negative	wild-type staining	30%	NA	24	Lung metastasis, death
5	Ates et al. (2024) (24)	30	pelvic pain and foul-smelling vaginal discharge,Long history LSIL	HPV6/11	4	Papillary,Well differentiated, invasive solid squamous nests	Negative	wild-type staining	positivity in the basal epithelial layer	NA	36	NED
6	Sun et al. (2025) (19)	34	Long history of HSIL	HPV6	3.5	papillary, nodular, or cauliflower-like appearance	Focally positive	increased expression	70%-80%, nearly full thickness	*NFE2L2,NOTCH1* mutation	12	NED
7	Sun et al. (2025) (19)	55	Postmenopausal bleeding; cervical mass	HPV6	4.5	papillary, nodular, or cauliflower-like appearance	Negative	wild-type staining	20%-30%, most basal and parabasal layer	*NFE2L2* mutation	5	alive with disease
8	Sun et al. (2025) (19)	43	Cervical squamous intraepithelial lesion	HPV6	2	papillary, nodular, or cauliflower-like appearance	Negative	increased expression	70%-80%, nearly full thickness	*pTERT, NOTCH1,NOTCH, NOTCH3* mutation	NA	NA
9	Sun et al. (2025) (19)	63	Postmenopausal bleeding; 5cm mass completely replacing the cervix	HPV6	4.7	papillary, nodular, or cauliflower-like appearance	Negative	increased expression	70%-80%, nearly full thickness	*pTERT,NFE2L2, NOTCH3* mutation	9	alive with disease
10	Sun et al. (2025) (19)	65	Postmenopausal bleeding	HPV6	NA	papillary, nodular, or cauliflower-like appearance	Positive (block pattern)	increased expression	60%-70%, nearly full thickness	pTERT, NFE2L2 mutation	13	died of disease
11	Sun et al. (2025) (19)	58	Long history of cervical HSIL, LSIL	HPV11	Entire cervix	papillary, nodular, or cauliflower-like appearance	Negative	increased expression	70%-80%, nearly full thickness	*pTERT*,*NOTCH1,CDKN2A* mutation	22	alive with disease
12	Present Case	55	vaginal bleeding	HPV6	3.0	Exophytic papillary	Negative	NA	60% positive in hotspot areas	*TP53, CDKN2A, TERT, LATS1* mutation	2	NED

HPV, human papillomavirus; HSIL, High-Grade Squamous Intraepithelial Lesion; LSIL, Low-Grade Squamous Intraepithelial Lesion; NED, no evidence of disease; NA, Not available.

Histologically, these tumors demonstrate verrucous papillary squamous epithelial proliferation with prominent fibrovascular cores and a predominantly exophytic architecture. Although the morphology may mimic condyloma acuminatum, the presence of marked cytological atypia, increased mitotic activity, impaired epithelial maturation, and focal stromal invasion supports a diagnosis of carcinoma (4, 8, 9). Compared with high-risk HPV–associated CSCC, HPV6-related tumors tend to be better differentiated, exhibit more prominent keratinization, show limited stromal invasion, and demonstrate a lower frequency of lymphovascular space invasion.

From an immunophenotypic perspective, diffuse strong P16 expression is a well-established surrogate marker for high-risk HPV infection (10), a phenomenon driven by the binding of the high-risk HPV E7 oncoprotein to retinoblastoma protein (pRB). This interaction triggers pRB degradation, E2F transcription factor activation, and subsequent overexpression of P16INK4A. In contrast, low-risk HPV subtypes (e.g., HPV6, HPV11) lack a functional pRB-binding motif in their E7 proteins, resulting in negative or focal/patchy P16 expression. Consistent with prior reports—where 5 of 6 low-risk HPV-associated CSCC cases documented negative P16 expression (8)—our case also exhibited absent P16 staining. Ki67 expression in this rare entity is highly heterogeneous: over half of HPV6-associated CSCC cases display a Ki67 proliferation index >60% (comparable to high-grade squamous intraepithelial lesions [HSIL]), while others show restricted basal/parabasal staining with an index of 20%–30% (8). The elevated Ki67 index observed in this case supports the malignant nature of the lesion.

Accurate pathological diagnosis of low-risk HPV–associated CSCC can be challenging, particularly in limited biopsy specimens, where invasive foci may be underrepresented. Thorough sampling and integration of histomorphology, immunohistochemistry, and HPV genotyping are therefore essential to avoid misclassification as benign condyloma acuminatum or conventional high-risk HPV–associated CSCC.

### 3.2 Distinctive carcinogenic mechanisms

Recent molecular studies increasingly support an indirect carcinogenic model for low-risk HPV–associated CSCC, in which chronic viral-induced epithelial proliferation creates a permissive genomic landscape for the accumulation of oncogenic host mutations. A well-documented case of HIV-positive patient with HPV11-associated condyloma acuminatum (44 years old, no somatic mutations) progressing to HSIL-like lesions (55 years old, *TERT/NOTCH1* mutations) and finally invasive carcinoma (58 years old, expanded mutation spectrum) illustrates this stepwise progression (8). Immunocompromised individuals are at heightened risk due to impaired immune surveillance, accelerating mutation accumulation.The present patient had no evidence of immunosuppressive disease, organ transplantation, or long-term immunosuppressive therapy.Although immunosuppression may accelerate this process, the present case illustrates that malignant transformation can also occur in immunocompetent individuals, possibly through prolonged epithelial proliferation and cumulative genomic instability.

#### 3.2.1 *TERT* Alterations

*TERT* is a proto-oncogene silenced in normal somatic cells. Activating *TERT* mutations are detected in multiple malignancies (e.g., melanoma, hepatocellular carcinoma, urothelial carcinoma) (11, 12) and are more frequent in non-HPV-associated than HPV-associated squamous cell carcinomas of the vulva, penis, and head/neck (13–15). In cervical cancer, *TERT* promoter mutations occur in 0%–21.4% of cases (16–18), but in 66.7% (4/6) of low-risk HPV-associated CSCC (2 cases with *pTERT* c.-124C>T, 2 with *pTERT* c.-146C>T) (19). The detection of *pTERT* c.-124C>T in our case further supports *TERT* mutations as a key driver of low-risk HPV-associated cervical carcinogenesis.

#### 3.2.2 *CDKN2A* gene abnormalities

*CDKN2A* is a core tumor suppressor gene encoding p16INK4A and p14ARF, regulating cell cycle progression and genomic stability. In high-risk HPV-associated lesions, E7-mediated pRB degradation induces epigenetic de-repression of *CDKN2A*, leading to P16 overexpression. In contrast, *CDKN2A* genetic abnormalities (e.g., mutations, deletions) underlie negative P16 expression in low-risk HPV-associated lesions. The *CDKN2A* c.238C>T nonsense mutation in our case (resulting in truncated non-functional protein) likely explains the negative P16 staining. *CDKN2A* alterations are common in HPV-negative head and neck squamous cell carcinoma (20), and *CDKN2A* methylation is linked to cervical carcinogenesis suggesting a conserved role in HPV-independent squamous cell carcinoma (21).

#### 3.2.3 *TP53* gene mutations

*TP53* mutations have been reported in a subset of low-risk HPV–associated CSCC. Based on the limited number of published cases, the estimated frequency ranges from approximately 20% to 40% (4, 8), although the total number of documented cases remains extremely small and no large-scale cohort studies are available.

Unlike high-risk HPV, HPV6 E6 does not induce p53 degradation, and its E7 protein lacks the ability to effectively disrupt the pRB pathway. Consequently, *TP53* mutations may arise as secondary events driven by chronic inflammation, persistent epithelial proliferation, and accumulated DNA damage. Somatic *TP53* alterations thus represent an alternative mechanism of p53 pathway inactivation and may function as key drivers of malignant transformation in this context.

This mechanism parallels that observed in HPV-independent differentiated vulvar intraepithelial neoplasia, which exhibits an 88% *TP53* mutation rate (22), and in HPV-independent endometrial squamous cell carcinoma, in which *TP53* mutations are nearly universal (23). The two *TP53* nonsense mutations identified in this case, p.(Y126*) and p.(S183*), albeit present at low variant allele frequencies (likely subclonal), may contribute to tumorigenesis.

From a diagnostic perspective, low-risk HPV–associated cervical squamous cell carcinoma may be underrecognized, particularly in limited biopsy specimens, due to its prominent exophytic architecture and morphological resemblance to condyloma acuminatum (24). In such settings, adjunctive diagnostic tools become essential. Aberrant p53 immunohistochemical staining patterns—either diffuse strong nuclear overexpression or complete absence (null pattern)—may indicate underlying *TP53* mutation and support a diagnosis of malignancy, especially in lesions lacking diffuse p16 expression. This contrasts with conventional high-risk HPV–associated carcinomas, in which *TP53* is typically functionally inactivated by viral E6 rather than by genetic mutation.

Furthermore, HPV *in situ* hybridization or RNA-based assays (e.g., RNAscope) can localize viral nucleic acids within tumor cells, thereby strengthening causal attribution and distinguishing true viral-driven transformation from incidental HPV colonization. In diagnostically challenging cases, comprehensive integration of histomorphology, immunohistochemistry, HPV genotyping, and molecular profiling may reduce misclassification and improve diagnostic accuracy.

#### 3.2.4 *LATS1* gene mutations

*LATS1* is a serine/threonine kinase and negative regulator of *YAP1* in the Hippo pathway, suppressing tumorigenesis via inhibiting proliferation and promoting apoptosis. *LATS1* inactivation (via loss of heterozygosity, deletions, mutations, or promoter methylation) is observed in soft tissue sarcoma, adenoid cystic carcinoma, breast cancer, and lung cancer (25). Three pathogenic *LATS1* mutations were detected in our case; however, their role in low-risk HPV-associated CSCC remains unclear due to limited research, warranting further functional studies.

Collectively, these alterations converge on pathways regulating cell cycle control, telomere maintenance, and genomic stability, underscoring a host mutation–driven mechanism of malignant transformation.

### 3.3 Clinicopathological implications

Among eight previously reported cases of low-risk HPV–associated squamous cell carcinoma, six occurred in the cervix and two in the vulva. Only one case was associated with HPV11 infection, whereas the remaining seven were HPV6-positive (8). Masuda and Liu et al. have also reported cases of HPV6-associated cervical squamous cell carcinoma or high-grade intraepithelial lesions (4, 9). The present case was likewise HPV6-positive, suggesting that HPV6 may have a stronger association with squamous malignant transformation than other low-risk HPV types. Nevertheless, this conclusion requires validation in larger cohorts.

With respect to prognosis, four of the six reported cases had unfavorable outcomes, including one death and three cases with regional or distant metastases (19). Another report described a patient with HPV6-associated cervical squamous cell carcinoma who died from pulmonary metastases two years after surgery (26). Masuda et al. reported a case of HPV6-associated low-grade intraepithelial lesion that progressed to cervical squamous cell carcinoma with lymph node and lung metastases after three years of follow-up (9). Although the present patient has shown no evidence of recurrence or metastasis during a relatively short postoperative follow-up of more than two months, the available literature suggests that HPV6-associated cervical squamous cell carcinoma, despite its rarity, may be associated with an unfavorable overall prognosis.

Current cervical cancer screening strategies primarily target high-risk HPV types, potentially leading to underrecognition of rare low-risk HPV–associated malignancies. Clinicians should maintain vigilance when evaluating long-standing or recurrent HPV6-associated condylomatous lesions, particularly in high-risk populations.

## 4 Conclusion

HPV6-associated cervical squamous cell carcinoma is a rare malignant entity with distinctive pathological, immunophenotypic, and molecular features. Its carcinogenesis appears to involve chronic viral-induced epithelial proliferation combined with the accumulation of host somatic mutations, rather than direct viral oncogene–driven transformation. Accurate diagnosis requires careful integration of morphology, immunohistochemistry, and molecular testing. Recognition of this rare subtype broadens the biological spectrum of cervical squamous cell carcinoma and supports the integration of genomic profiling into the classification of atypical HPV-associated cervical tumors.

## Data Availability

The datasets presented in this study can be found in online repositories. The names of the repository/repositories and accession number(s) can be found in the article/supplementary material.
